# The effect of physically or non-physically forced sexual assault on trajectories of sport participation from adolescence through young adulthood

**DOI:** 10.1186/s13690-020-00435-w

**Published:** 2020-06-10

**Authors:** Chung Gun Lee, Junhye Kwon, Hojun Sung, Inae Oh, Ohsup Kim, Jeehyun Kang, Ji-Won Park

**Affiliations:** 1 Department of Physical Education, College of Education, Seoul National University, 1 Gwanak-ro, Gwanak-gu, Seoul, 08826 South Korea; 2 Department of Taekwondo, College of Sports Science, Woosuk University, 443 Samnye-ro, Samnye-eup, Wanju-gun, Jeollabuk-do 55338 South Korea

**Keywords:** Sexual assault, Sport participation, Group-based trajectory modeling, Adolescent, Young adult

## Abstract

**Background:**

Sexual assault is one of potential factors that may greatly affect an individual’s sport participation. The purpose of this study is to investigate the effect of experiencing physically or non-physically forced sexual activity on trajectories of sport participation from adolescence to young adulthood.

**Methods:**

This study used the National Longitudinal Study of Adolescent Health (Add Health) data. Group-based trajectory modeling was utilized to examine the effect of experiencing sexual assault on trajectories of sport participation from adolescence to young adulthood.

**Results:**

A three-group trajectory model (high-stable group, high-decreasing group, and low-stable group) best fit sport participation among male participants and a two-group trajectory model (high-decreasing group and low-stable group) best fit sport participation among female participants. Both physically and non-physically forced sexual activity did not have significant effect on trajectories of sport participation among male participants. On the other hand, non-physically forced sexual assault significantly affected sport participation trajectory among female participants.

**Conclusions:**

Special care is required in developing sport promotion program for women victims of non-physically forced sexual activity. The results of this study also suggest that group-based trajectory modeling is a useful technique to examine distinct trajectories of sport participation from adolescence through young adulthood.

## Background

Engaging in regular physical activity has been shown to reduce the risk of developing chronic diseases, such as coronary heart disease, stroke, hypertension, various types of cancer, Type 2 diabetes mellitus, and osteoporosis [1]. Performing regular physical activity also has been shown to have beneficial effects on psychological well-being through enhancing self-esteem and reducing psychological distress [1]. Despite the known health benefits of regular physical activity, the prevalence of physical activity among adults (aged 18 or older) has been still unsatisfactory [2]. One of the most efficient ways to promote regular physical activity is through sport participation because participating in sports makes physical activity more enjoyable through competition, social interaction, goal achievement, and personal challenge [3]. Moreover, most sporting activities easily meet recent physical activity recommendations suggested by American College of Sports Medicine (ACSM) [4]. It is, therefore, important to investigate the factors that may influence sport participation because participation in sporting activities almost always involves physical activity.

Life change events, such as obtaining a new job, a change in marital status, parental incarceration, experiencing sexual assault, pregnancy, and the death of a family member, can be defined as “those occurrences, including social, psychological and environmental, which require an adjustment or effect a change in an individual’s pattern of living.” [5]. Such events are potential factors that may greatly affect an individual’s physical activity-related behaviors (e.g., sport participation, exercise, and outdoor recreation). Although life change events may affect a person’s commitment to be physically active by creating emotional distress and disturbing an individual’s daily routine, the effect of life change events on physical activity-related behaviors is poorly studied [6]. Certain life change events were shown to have strong effects on physical activity-related behavior but more research is needed to investigate the longer-term effects of different life change events on physical activity-related behaviors [6]. Among various life change events, this study focused on experiencing sexual assault.

A recent study showed that 23.4% of men and 43.9% of women have experienced various forms of sexual violence, such as being made to penetrate, non-physically pressured unwanted penetration, and kissing or fondling, during their lifetimes in the United States [7]. Breiding et al. also reported that 1.7% of men and 19.3% of women have experienced completed rape at some point in their lives [7]. Sexual assault is a serious public health problem especially for women because experiencing sexual assault places women at risk of various deleterious physical and psychological health outcomes, including depressive symptoms, suicidal ideation, posttraumatic stress disorder (PTSD) symptoms, anxiety, greater health care utilization, self-reported health complaints, and chronic health conditions [8–13]. These negative effects of sexual assault on psychological and physical health are known to persist for long periods of time [8, 14, 15].

Moreover, experiencing sexual assault is also associated with health-related behaviors, such as sexual intercourse as an affect regulation strategy [16] and problematic drinking [17–19]. The predominant hypothesis about this association is that sexual assault survivors engage in risky behaviors that are intended to cope with the psychological distress that results from their experience of sexual abuse, but which impair their physical health. Ullman et al. found that posttraumatic stress disorder (PTSD) symptoms mediated the effect of child sexual abuse on problematic drinking among adult women [20]. Goldstein et al. (2010) also found that among college students the motives for drinking (i.e., coping with depression) mediated the relation between childhood abuse and consequences of drinking. Although there are several studies that investigated the effect of experiencing sexual assault on health-related behaviors, few studies have considered psychological distress as a potential mediator of this relationship and, to our knowledge, none of the studies examined physical activity-related behaviors among victims of sexual assault. Since sexual assault survivors are likely to be harmed by societal stereotypes that aggravate victim blaming [21], it is possible that impaired mental health caused by societal stereotypes may limit survivors’ physical activity or sport participation.

Although some evidence supports the notion that experiencing sexual assault can affect physical activity-related behaviors, the extent and nature of these relationships across the lifespan are unclear. The National Longitudinal Study of Adolescent Health (Add Health), which prospectively followed up a nationally representative sample of middle and high school students in the United States [22], provides an opportunity to explore the effects of life change events on trajectories of sport participation from adolescence through young adulthood. The purpose of this study is to investigate the effect of experiencing physically or non-physically forced sexual activity on trajectories of sport participation from adolescence to young adulthood. We investigated separately by gender because women may be deleteriously affected by sexual assault in a different way or to a different degree than men [23–25] and it has been shown that men engage in higher levels of physical activity than women [2]. We also considered psychological depression as a mediator of the relationship between experiencing sexual assault and sport participation because previous studies supported the importance of psychological distress as a potential mediator of this relationship [16, 20, 26].

## Methods

### Data

This study used the National Longitudinal Study of Adolescent Health (Add Health) data. Add Health is a four-wave longitudinal study that prospectively followed up a nationally representative sample of 7th through 12th grade students (middle and high school students) in the United States. All public and private high schools in the United States that included more than 30 students were included in the initial sampling frame and these high schools were stratified by ethnic mix, size, urbanicity, region, and school type. Eighty high schools were then selected using systematic random sampling and approximately 70% of these schools were recruited. All public and private middle schools in the United States that sent graduates to recruited high schools were also recruited. A total of 134 schools were included in the final sample. In each school, students were randomly selected from official school rosters after stratification for age and grade. Wave 1 in-home survey was conducted from April to December in 1995 (*n* = 20,745). The first follow-up survey (wave 2) was conducted approximately 1 year later (*n* = 14,738). Wave 3 (*n* = 15, 197) and 4 (*n* = 15,701) in-home survey were conducted 6 and 12 years after wave 1, respectively. Further information about Add Health is reported elsewhere [22]. The present study used wave 1 through 4 public-use datasets (*n* = 6504).

### Measures

Sport participation at each wave was assessed via a question, “During the past week, how many times did you play an active sport, such as baseball, softball, basketball, soccer, swimming, or football?” According to the American College of Sports Medicine (ACSM), people need to engage in moderate to vigorous physical activity at least 30 min per day on 5 days per week in order to maintain their health [27]. Therefore, a participant who participated in sports 5 or more times during the past week defined as an active participator. Because sport participation at wave 3 and 4 was assessed by asking two questions, “In the past seven days, how many times did you participate in individual sports such as running, wrestling, swimming, cross-country skiing, cycle racing, or martial arts?” and “In the past seven days, how many times did you participate in strenuous team sports such as football, soccer, basketball, lacrosse, rugby, field hockey, or ice hockey?”, the number of times participated in team and individual sports were combined to create a total number of participation in sports.

Physically forced sexual activity in each wave was assessed in wave 4 via two questions, “Have you ever been physically forced to have any type of sexual activity against your will?” and “How old were you the first or only time this happened?” If a participant was physically forced to have sexual activity before each wave of the survey, he or she was defined as a participant who experienced physically forced sexual assault. Non-physically forced sexual activity in each wave was assessed in wave 4 by asking two questions, “Have you ever been forced, in a non-physical way, to have any type of sexual activity against your will? For example, through verbal pressure, threats of harm, or by being given alcohol or drugs?” and “How old were you the first or only time this happened?” If a participant was non-physically forced to have sexual activity before each wave of the survey, he or she was defined as a participant who experienced non-physically forced sexual abuse.

A short version of the Center for Epidemiologic Studies-Depression Scale (CES-D) was utilized to evaluate participants’ psychological depression at each wave [28, 29]. After reverse coding several items, depression scores for each item were summed to indicate psychological depression at each wave.

### Statistical analysis

The present study utilized group-based trajectory modeling to examine the effect of experiencing sexual assault on trajectories of sport participation from adolescence to young adulthood. Since sport participation is a binary variable, binary logit models were fitted to estimate trajectories of sport participation using SAS Traj procedure [30]. The wave was used as a time scale (wave 1 = 1996, wave 2 = 1997, wave 3 = 2001, and wave 4 = 2008) and was centered at wave 1 (wave 1 = 0, wave 2 = 1, wave 3 = 6, and wave 4 = 12). The number of trajectory groups and the shape of each trajectory group were selected based on statistical [31] and non-statistical criteria [32]. These criteria for identifying the best fitting model included the Bayesian Information Criterion (BIC), average posterior probabilities of group assignment, and percentages of observations assigned to each group. Subjective judgment is also used in model selection because it is important to identify useful and parsimonious model [32]. Once the best fitting model was identified, time-varying covariates were added into the model. The purpose of this modeling extension is to examine the effect of physically or non-physically forced sexual activity on sport participation trajectories [33]. The function called “tcov” is used to direct SAS Traj procedure to calculate the trajectory for each group under an author-specified set of values of time-varying covariate. The command “plottcov 1 1 1 1” was also used to direct SAS Traj procedure to estimate the predicted trajectories under the assumption that experiencing sexual assault equals 1 in all waves. This function allows us to see sport participation trajectories for those who experienced sexual assault before wave 1. Psychological depression was also included in the model as time-varying covariate because it is a potential mediator of the relationship between experiencing sexual assault and sport participation. Characteristics of participants were presented as frequency and percent for categorical variables and mean and standard deviation for continuous variables. Comparisons between trajectory groups were performed using chi-square test for categorical variables and analysis of variance (ANOVA) for continuous variables. All analyses were performed using SAS version 9.4 (SAS Institute Inc., Cary, NC).

## Results

### Descriptive statistics

Table 1 shows characteristics of participants by sport participation trajectory group. The mean age of male participants was 15.61 years at wave 1. Male participants were divided into three sport participation trajectory groups (i.e., low-stable group, high-decreasing group, and high-stable group). The low-stable group consistently had low probability of sport participation from adolescence to young adulthood. The remaining two groups both started with high probability of participating in sports, one maintaining that pattern until they become young adult (high-stable group), the other being less likely to participate in sports with time (high-decreasing group). Low-stable group had the highest mean depression score and high-stable group had the lowest mean depression score. At each wave, the prevalence of experiencing physically and non-physically forced sexual assault was similar across groups. Table 1 also represents characteristics of female participants by sport participation trajectory group. Unlike male participants, there were only two sport participation trajectory groups in female participants (i.e., low-stable group and high-decreasing group). The mean age of female participants at wave 1 (15.46 years) was similar to that of male participants. Low-stable group had higher mean depression score than high-decreasing group at each wave. The prevalence of experiencing physically and non-physically forced sexual activity was similar between two trajectory groups.

**Table 1:** Characteristics of participants by sport participation trajectory group (*N* = 3147)

Characteristics	Total sample	Sport participation trajectory groups			*p*-value
		Low-stable	High-decreasing	High-stable	
Male participants					
Mean age at wave 1 (*SD*)	15.61 (1.79)	15.92 (1.79)	15.27 (1.71)	15.12 (1.74)	<.0001
Mean depression (*SD*)					
Wave 1	4.33 (3.29)	4.68 (3.41)	4.00 (3.12)	3.64 (3.03)	<.0001
Wave 2	4.20 (3.26)	4.48 (3.43)	3.99 (3.12)	3.72 (2.87)	0.0001
Wave 3	3.50 (3.13)	3.52 (3.09)	3.65 (3.30)	2.99 (2.74)	0.0097
Wave 4	4.29 (3.25)	4.40 (3.32)	4.27 (3.17)	3.82 (3.10)	0.0289
Ever forced to have sex in a non-physical way (%)					
Wave 1	42 (1.79)	21 (1.64)	18 (2.26)	3 (1.09)	0.3828
Wave 2	46 (1.96)	22 (1.72)	20 (2.51)	4 (1.46)	0.3667
Wave 3	63 (2.68)	28 (2.19)	28 (3.52)	7 (2.55)	0.1886
Wave 4	82 (3.49)	40 (3.13)	33 (4.15)	9 (3.28)	0.4609
Ever forced to have sex in a physical way (%)					
Wave 1	34 (1.45)	16 (1.25)	13 (1.64)	5 (1.82)	0.6597
Wave 2	34 (1.45)	16 (1.25)	13 (1.64)	5 (1.82)	0.6597
Wave 3	38 (1.62)	19 (1.48)	14 (1.76)	5 (1.82)	0.8488
Wave 4	43 (1.83)	22 (1.72)	15 (1.89)	6 (2.19)	0.8583
Sport participation ≥5 times per week (%)					
Wave 1	1067 (33.94)	0 (0.00)	849 (75.60)	218 (71.01)	<.0001
Wave 2	781 (33.75)	0 (0.00)	607 (65.48)	174 (67.18)	<.0001
Wave 3	273 (12.17)	59 (4.91)	0 (0.00)	214 (76.70)	<.0001
Wave 4	218 (9.27)	61 (4.76)	0 (0.00)	157 (57.30)	<.0001
Total (%)	3147	1716 (46.39)	1123 (37.46)	308 (16.15)	
Female participants					
Mean age at wave 1 (*SD*)	15.46 (1.78)	15.72 (1.76)	14.69 (1.62)		<.0001
Mean depression (*SD*)					
Wave 1	5.43 (3.89)	5.66 (3.96)	4.74 (3.59)		<.0001
Wave 2	5.57 (3.94)	5.71 (3.94)	5.22 (3.92)		0.0051
Wave 3	4.33 (3.80)	4.40 (3.80)	4.16 (3.78)		0.1514
Wave 4	5.09 (3.77)	5.20 (3.80)	4.78 (3.65)		0.0113
Ever forced to have sex in a non-physical way (%)					
Wave 1	224 (8.15)	181 (8.80)	43 (6.20)		0.0306
Wave 2	260 (9.46)	205 (9.97)	55 (7.94)		0.1135
Wave 3	416 (15.13)	318 (15.47)	98 (14.14)		0.3998
Wave 4	547 (19.90)	415 (20.18)	132 (19.05)		0.5167
Ever forced to have sex in a physical way (%)					
Wave 1	166 (6.02)	131 (6.35)	35 (5.05)		0.2135
Wave 2	183 (6.64)	143 (6.93)	40 (5.77)		0.2888
Wave 3	300 (10.89)	224 (10.86)	76 (10.97)		0.9366
Wave 4	386 (14.01)	291 (14.11)	95 (13.71)		0.7944
Sport participation ≥5 per week (%)					
Wave 1	515 (15.35)	0 (0.00)	515 (62.12)		<.0001
Wave 2	422 (16.75)	0 (0.00)	422 (58.29)		<.0001
Wave 3	134 (5.12)	0 (0.00)	134 (19.45)		<.0001
Wave 4	115 (4.17)	62 (3.00)	53 (7.65)		<.0001
Total (%)	3356	2527 (68.30)	829 (31.70)		

Missing data were excluded in calculating the percentage

### Group-based trajectory modeling

**Fig. 1:**
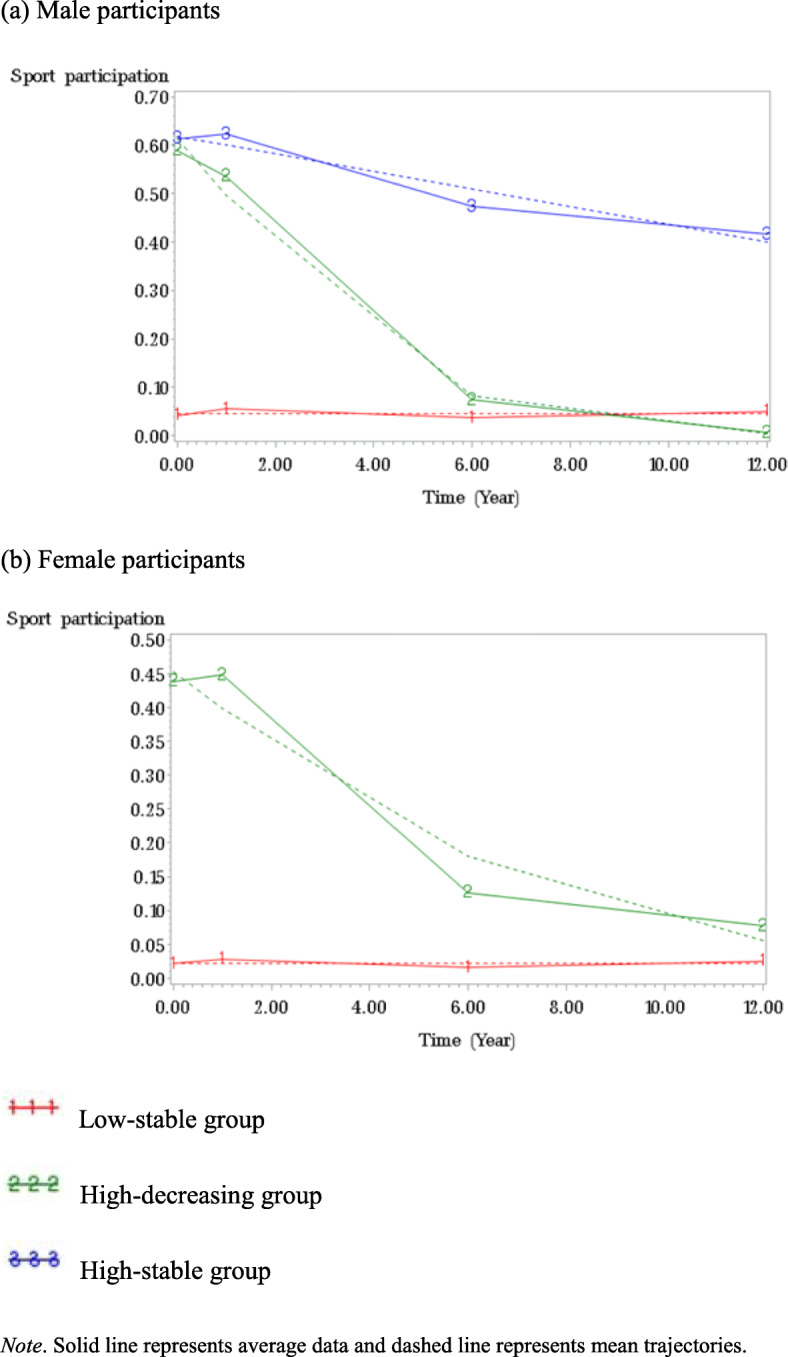
Trajectories of sport participation from adolescence through young adulthood. **a** Male participants. **b** Female participants.  Low-stable group.  High-decreasing group.  High-stable group. Note. Solid line represents average data and dashed line represents mean trajectories

A three-group trajectory model best fit sport participation among male participants (see Fig. 1 and Table 2). About half (46.39%) of male participants were assigned to low-stable group. The remaining male participants started with high probability of participating in sports, 16.15% of them maintaining that pattern until they become young adult (high-stable group), 37.46% of them being less likely to participate in sports with time (high-decreasing group). A two-group trajectory model best fit sport participation among female participants (see Fig. 1 and Table 2). Most female participants (68.30%) were assigned to low-stable group. The remaining female participants (31.70%) had high probability of sport participation during adolescence and then it decreased until they become young adult.

**Table 2:** Bayesian information criterion for selection of models

Gender	Model	Number of groups	Order	BIC
Men	1	4	2, 2, 2, 2	− 4912.57
	2	3	0, 1, 1	− 4894.60
	3	3	2, 2, 2	− 4898.28
	4	2	2, 2	− 4908.40
	5	1	2	− 5074.67
Women	1	3	2, 2, 2	− 3498.00
	2	2	0, 1	−3498.61
	3	2	2, 2	− 3493.88
	4	1	2	− 3609.69

The order shows whether trajectories were fit with constant (0), linear (1), or quadratic (2). *BIC* bayesian information criterion

**Fig. 2:**
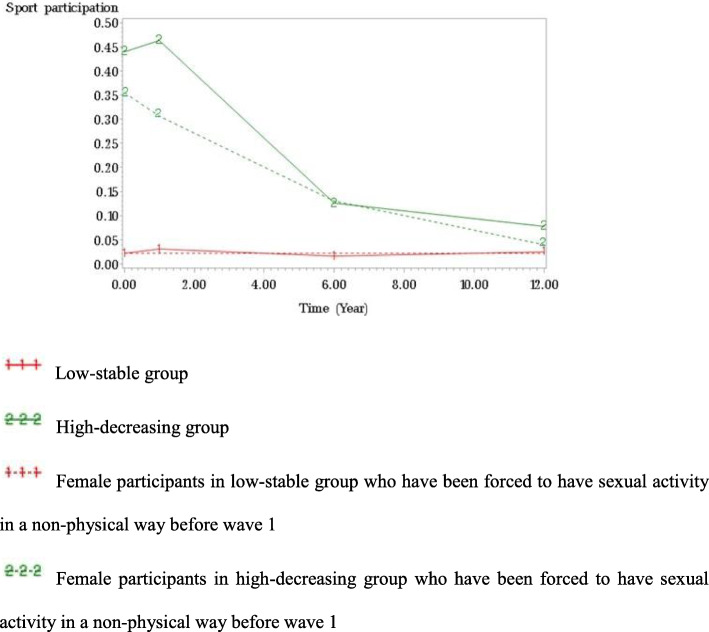
Effect of non-physically forced sexual activity (ever) on trajectories of sport participation from adolescence through young adulthood among female participants.  Low-stable group.  High-decreasing group.  Female participants in low-stable group who have been forced to have sexual activity in a non-physical way before wave 1.  Female participants in high-decreasing group who have been forced to have sexual activity in a non-physical way before wave 1

Tables 3 and 4 represent the effect of experiencing physically and non-physically forced sexual activity on sport participation trajectories, respectively. Both physically and non-physically forced sexual activity did not have significant effect on sport participation among male participants. On the other hand, non-physically forced sexual assault significantly affected sport participation among female participants. Female participants who experienced non-physically forced sexual activity were significantly less likely to participate in sports throughout the trajectory in high-decreasing group (see Fig. 2). However, the significant effect of non-physically forced sexual activity on women’s sport participation became insignificant after controlling for psychological depression.

**Table 3:** The effect of physically (a) or non-physically (b) forced sexual activity on sport participation trajectories among male participants (*N* = 3147)

Group	Parameter	Model 1		Model 2	
		Estimate	(*SE*)	Estimate	(*SE*)
(a) Physically forced sexual activity					
1	Intercept	−3.082	(0.196)***	−3.026	(0.276)***
	Ever forced to have sex in a physical way	0.112	(1.012)	0.128	(1.169)
	Depression			−0.023	(0.049)
2	Intercept	0.463	(0.120)***	0.768	(0.160)***
	Linear	−0.475	(0.069)***	−0.504	(0.072)***
	Ever forced to have sex in a physical way	0.494	(0.586)	0.689	(0.591)
	Depression			−0.082	(0.026)**
3	Intercept	0.473	(0.167)**	0.630	(0.188)***
	Linear	−0.074	(0.033)*	−0.075	(0.029)*
	Ever forced to have sex in a physical way	−0.148	(0.624)	0.014	(0.606)
	Depression			−0.055	(0.024)*
(b) Non-physically forced sexual activity					
1	Intercept	−3.083	(0.196)***	−3.021	(0.273)***
	Ever forced to have sex in a physical way	0.496	(0.663)	0.608	(0.694)
	Depression			−0.020	(0.047)
2	Intercept	0.446	(0.120)***	0.760	(0.160)***
	Linear	−0.473	(0.068)***	−0.505	(0.071)***
	Ever forced to have sex in a non-physical way	0.594	(0.488)	0.824	(0.501)
	Depression			−0.085	(0.026)***
3	Intercept	0.491	(0.166)**	0.652	(0.187)***
	Linear	−0.073	(0.032)*	−0.076	(0.028)**
	Ever forced to have sex in a non-physical way	−1.003	(0.677)	−0.916	(0.682)
	Depression			−0.053	(0.024)*

* *p* < .05, ** *p* < .01, *** *p* < .001

**Table 4:** The effect of physically (a) or non-physically (b) forced sexual activity on sport participation trajectories among female participants (N = 3356)

Group	Parameter	Model 1		Model 2	
		Estimate	(*SE*)	Estimate	(*SE*)
(a) Physically forced sexual activity					
1	Intercept	−3.721	(0.197)***	−3.809	(0.321)***
	Ever forced to have sex in a physical way	−0.401	(0.560)	−0.300	(0.550)
	Depression			0.016	(0.042)
2	Intercept	−0.154	(0.122)	0.042	(0.144)
	Linear	−0.217	(0.017)***	−0.219	(0.017)***
	Ever forced to have sex in a physical way	−0.239	(0.216)	−0.151	(0.225)
	Depression			−0.047	(0.017)**
(b) Non-physically forced sexual activity					
1	Intercept	−3.733	(0.208)***	−3.877	(0.341)***
	Ever forced to have sex in a non-physical way	−0.049	(0.393)	0.001	(0.407)
	Depression			0.026	(0.041)
2	Intercept	0.131	(0.124)	0.056	(0.145)
	Linear	−0.216	(0.017)***	−0.217	(0.018)***
	Ever forced to have sex in a non-physical way	−0.469	(0.207)*	−0.412	(0.217)
	Depression			−0.047	(0.017)**

* *p* < .05, ** *p* < .01, *** *p* < .001

## Discussion

To our knowledge, this is the first study that examined the effect of physically or non-physically forced sexual activity on trajectories of sport participation from adolescence through young adulthood using group-based trajectory modeling. In Add Health data, stratified sampling was utilized with the purpose of gathering data that is inclusive of diverse racial backgrounds and genders. Male participants were divided into three trajectory groups. The largest group (46.39%) consistently had low probability of sport participation from adolescence to young adulthood. The remaining two groups both started with high probability of participating in sports, one (16.15%) maintaining that pattern until they become young adult, the other (37.46%) being less likely to participate in sports with time. On the other hand, females were divided into only two trajectory groups. The majority group (68.30%) steadily had low probability of participating in sports throughout the study period. The other group (31.70%) had high probability of sport participation at the beginning and then it decreased over time. An interesting finding is that there is no “low-increasing group” in both gender groups. This result is in line with the previous studies suggesting that overall prevalence of physical activity or sport participation decreases during the transition from adolescence to adulthood [34] and, therefore, participation in sports or any type of physical activity during adolescence can never be emphasized enough [35]. School physical education and other programs affecting physical activity-related behaviors among adolescents should strive to develop and implement efficient physical activity programs. Moreover, future studies should be conducted to find out factors that influence sustainable physical activity behavior that continues throughout the lifespan especially for females because there was no “high-stable group” among female participants in this study.

The main finding of this study is that women who experienced non-physically forced sexual activity were significantly less likely to participate in sports throughout the trajectory in “high-decreasing group”. An insignificant effect of non-physically forced sexual activity on sport participation trajectories among men may be explained as follows. First, preliminary data suggest that women are more likely than men to be deleteriously affected by sexual assault [23–25]. Therefore, women victims of sexual assault may experience higher levels of psychological distress that can hamper their efforts to participate in sports. Second, extremely small samples of male victims (only 82 men experienced non-physically forced sexual activity) disguised the statistic results. Given the paucity of research on sexual assault in men, more research is needed on how sex differences interact with experiencing sexual assault to influence sport participation.

One interesting finding is that only non-physically forced sexual activity had significant effect on sport participation trajectory among women. According to attribution theory, perceived freedom to act is positively related to perceived internality [36]. If assailant and victim have engaged in voluntary social contact and if sexual assault does not involve physical force, blame and responsibility may be attributed to the victim and, therefore, some type of internal attribution about the victim can be made [37]. These suggest that victims whose assailants had used little or no force may experience more severe adjustment problems because they are especially prone to self-blame [37, 38]. Moreover, in a dating relationship of young people, non-physically forced sexual assault is actually shown to be perceived as socially permissible [39, 40]. In our study, it is possible that severe adjustment problems caused by societal stereotypes that exacerbate victim blaming may have impaired victims’ efforts to engage in sport participation among women who experienced non-physically forced sexual activity.

The effect of non-physically forced sexual activity on women’s sport participation was considerably weakened and became insignificant after including psychological depression in the model as a potential mediator. This result partially supports our hypothesis which asserts that sexual assault victims engage in health risk behaviors because psychological distress caused by experiencing sexual assault hampers their efforts to promote their health. This result is also in line with previous studies showing that psychological distress mediated the effect of experiencing sexual assault on various health-related behaviors among women [16, 20, 26]. Further studies are needed to examine other potential mediators in addition to psychological depression when examining the effect of non-physically forced sexual activity on sport participation in women.

This study has several important limitations. First, since more specific information about sport participation was not available in Add Health data, individual sport participation could, therefore, not be distinguished from team sport participation. Moreover, only the number of days of participating in sports was considered in assessing sport participation. To understand more specifically the influence of experiencing sexual assault on sport participation, future studies need to use more precise measure of sport participation. Second, the small number of waves may have affected group membership and shape of trajectory. Only linear trajectory shapes appeared to be statistically significant, maybe because Add Health data had only four waves. Third, all the variables used in this study were measured by self-report questionnaires, which could introduce response bias or recall bias. Lastly, since this study analyzed a secondary dataset (Add Health data), it was impossible to consider other factors that may have contributed to the relationship between sexual assault and sport participation.

## Conclusion

Despite these limitations, the findings of this study can be contributable to the literature by providing critical information on the effect of experiencing non-physically forced sexual activity on women’s sport participation trajectories from adolescence through young adulthood. Special care is required in developing sport promotion program for women victims of non-physically forced sexual activity. The results of this study also suggest that group-based trajectory modeling is a useful technique to examine distinct trajectories of sport participation from adolescence through young adulthood.

## Data Availability

The datasets generated and/or analyzed during the current study are available in the [Add Health The National Longitudinal Study of Adolescent to Adult Health] repository, [https://www.cpc.unc.edu/projects/addhealth].

## Abbreviations

ACSM: American College of Sports Medicine
Add health: National Longitudinal Study of Adolescent Health
CES-D: Center for Epidemiologic Studies-Depression Scale
BIC: Bayesian Information Criterion
SAS: Statistical Analysis System
ANOVA: Analysis of Variance
